# Safety and efficacy of wiping lid margins with lid hygiene shampoo using the “eye brush”, a novel lid hygiene item, in healthy subjects: a pilot study

**DOI:** 10.1186/s12886-019-1052-y

**Published:** 2019-02-04

**Authors:** Hirotaka Tanabe, Motoko Kawashima, Minako Kaido, Reiko Ishida, Tetsuya Kawakita, Kazuo Tsubota

**Affiliations:** 1 Department of Ophthalmology, Keio University School of Medicine, 35 Shinanomachi, Shinjuku-ku, Tokyo, 160-8582 Japan; 2 Department of Ophthalmology, Ashikaga Red Cross Hospital, 284-1, Yobecho, Ashikaga, Tochigi 326-0843 Japan; 3 Wada Eye Clinic, 2578-27, Hojo, Tateyama, Chiba 294-0045 Japan

**Keywords:** Lid hygiene, Meibomian gland dysfunction, MGD, Eye shampoo, Dry eye, Eye brush

## Abstract

**Background:**

To evaluate the safety and efficacy of using a lid hygiene brush prototype to wipe the lid margins with lid hygiene shampoo in subjects with normal meibomian glands.

**Methods:**

Twelve eyes of 6 subjects were all evaluated just before and after wiping lid margins using 1) tap water alone, 2) Eye Shampoo, 3) Eye Brush, or 4) both products, each during a different week. The results after using both products twice daily for 1 month were also evaluated. Wiping efficacy was determined by post-wiping scores for the remaining fluorescein-stained 0.3% Tarivid ointment fully applied to eyelids and lid margins under microscopic view illuminated by blue light just after performing each of the four lid hygiene methods described above.

**Results:**

No significant deterioration in ocular conditions occurred. Eyestrain, eye discharge, and dryness decreased with tap water (*P* = 0.020), Eye Shampoo (*P* = 0.036), and Eye Brush (*P* = 0.014), respectively. Sensations of eye discharge increased after 1 month of using both products (*P* = 0.042). The wiping efficacy of Eye Brush, Eye Shampoo or both was significantly greater than that of tap water alone (two-sided test, *P* = 0.003, 0.003, 0.002), and using both significantly increased efficacy above Eye Shampoo use alone (one-sided test, *P* = 0.009).

**Conclusions:**

Wiping lid margins using Eye Brush enhanced the cleansing power of Eye Shampoo. A daily healthcare routine using both products could be a safe and effective option for daily lid hygiene.

**Trial registration:**

UMIN000016905. Registration date: March 24, 2015; the study was prospectively registered.

**Electronic supplementary material:**

The online version of this article (10.1186/s12886-019-1052-y) contains supplementary material, which is available to authorized users.

## Background

In recent years, several lid hygiene products have emerged for the prevention or treatment of ocular diseases in response to the increased prevalence of new eye cosmetics and an improved understanding of the importance of lid hygiene [1–7]. Eye Shampoo (MediProduct Co., Ltd., Tokyo, Japan) is one of those options [6, 7]. We developed a lid hygiene brush prototype (Eye Brush) similar to a toothbrush and evaluated the safety and efficacy of using it in conjunction with lid hygiene shampoo (Eye Shampoo) through a pilot study involving healthy subjects.

## Methods

### Eye brush prototype

**Fig. 1:**
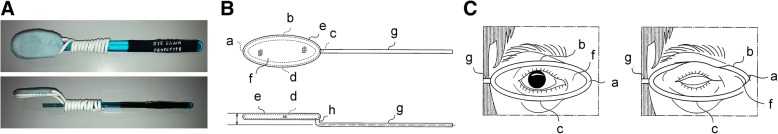
Structure of the Eye Brush prototype (a/b) and how to use it (c). a: Top view and side view, b: Top view and side view, c: Before use and during use: a. curved section, b. first abutting part, c. curved section, d. second abutting part, e. wiping material, f. space, g. gripper, h. bending section of the Eye Brush prototype (b and c)

There is a need for a tool that allows for the simple and efficient cleaning of the eyelid margin and specifically, a tool that can be used to prevent or treat meibomian gland dysfunction (MGD). The eyelid margin cleaning tool includes a wiping material with a surface capable of cleaning the user’s eyelid margin, a support to which the wiping material is attached, and a gripper attached to the support. The support is equipped with two abutting parts that make contact with the surfaces of the user’s upper (1st part) and lower (2nd) eyelids through the wiping material. These two abutting parts are arranged apart from each other to form a space that can accept the deflected wiping material. The eyeball can be moderately moved backward, and either the upper or lower eyelid can be everted to expose the eyelid margin surface to the surface of the wiping material by placing the abutting parts of the support to which the wiping material is attached on the user’s upper and lower eyelid surfaces and pressing the surfaces with the abutting parts. Accordingly, the surface of the eyelid margin can be efficiently cleaned without touching the eyeball with the wiping material. The support contains curved sections connecting at least one side of the longitudinal ends of the two abutting parts, and the wiping material is attached to the curved section. The region around the eyelid margin, including the inner and outer canthi, can be easily cleaned with the surface of the wiping material attached to the curved section. The support is oval in shape along the contour of the eyeball, and the surface of the eyelid margin can be easily everted along this contour. The gripper is attached to the longitudinal end of the support, and the plane demarcated by the two abutting parts is substantially deviated from the longitudinal axis of the gripper in parallel with it. Because of this design, users can easily repeat lateral movements of the gripper attached to the support in the longitudinal direction and clean the surfaces of their eyelid margins. Since the plane demarcated by the two abutting parts is substantially deviated from the longitudinal axis of the gripper and parallel to it, the user’s nose does not interfere with the repeated lateral movement of the eyelid margin cleaning tool (Fig. 1a and b).

### Eye shampoo

We used Eye Shampoo (MediProduct Co., Ltd., Tokyo, Japan) as a lid hygiene product in this pilot study, and its characteristics and ingredients are shown in Table 1.

**Table 1:** Eye shampoo (MediProduct) 1) Well-balanced cleansing ingredients are blended for less irritation and a high cleansing efficacy. 2) Osmotic pressure: 300 mOsm/L; pH: 7.4 (similar to the pH of tears). 3) Contains moisturizing and anti-inflammatory ingredients

Eye shampoo ingredients	Function
WATER; AQUA	Solvent
POLYGLYCERYL-4 LAURYL ETHER	Surfactant-Cleansing Agent
SODIUM CHONDROITIN SULFATE	Conditioning Agent-Humectant
SODIUM HYALURONATE	Conditioning Agent-Humectant
DIPOTASSIUM GLYCYRRHIZATE	Conditioning Agent-Humectant
ALLANTOIN	Conditioning Agent-Humectant
PANTHENOL	Conditioning Agent-Humectant
CALCIUM CARBONATE	Buffering Agent
SODIUM POLYACRYLATE	Viscosity Increasing Agent
SODIUM CHLORIDE	Viscosity Increasing Agent
POTASSIUM CHLORIDE	Buffering Agent
XANTHAN GUM	Viscosity Increasing Agent
PROPANEDIOL	Conditioning Agent-Humectant
CARBOMER	Viscosity Increasing Agent
POTASSIUM HYDROXIDE	pH Adjuster
HYDROXYPROPYL CYCLODEXTRIN	Emulsion Stabilizer
IODOPROPYNYL BUTYLCARBAMATE	Preservative
PHENOXYETHANOL	Preservative

### Procedure details

#### Tap water

Eyelids were washed with tap water for 30 s, keeping the eyes lightly closed.

#### Eye shampoo

Shampoo was pumped onto one hand, spread gently around the eyes and lightly massaged onto eyelids to remove impurities located at the eyelash roots. The eyelids were rinsed with tap water for 30 s, keeping the eyes lightly closed.

#### Eye brush

The brush was placed gently onto the eyelids and used to lightly massage them for 30 s to remove impurities located at the eyelash roots. Then, the eyelids were rinsed with tap water for 30 s, keeping the eyes lightly closed.

#### Eye shampoo and eye brush

Shampoo was pumped onto the eye brush, and the brush was placed gently onto the eyelids and used to lightly massage them for 30 s to remove impurities located at the eyelash roots. The eyelids were rinsed with tap water for 30 s, keeping the eyes lightly closed.

### Study subjects and protocol

Twelve eyes of 6 subjects (6 males aged 32–56 [39.8 ± 9.62] years) with normal meibomian glands were evaluated before and after wiping the lid margins in the following ways: using tap water alone, using Eye Shampoo, using the Eye Brush, or using both Eye Shampoo and the Eye Brush. Each procedure was performed during a different week, and the evaluation was performed just after the procedure. The results after using both Eye Shampoo and the Eye Brush twice daily for 1 month were also evaluated. Scores related to ocular condition, i.e., the tear break-up time (TBUT) (graded on a scale of 0–10), corneal and conjunctival fluorescein/lissamine green/rose bengal staining scores (graded 0–9), lid-margin lissamine green staining scores (graded 0–3), subjective symptoms (graded 0–100, as assessed via a visual analog scale [VAS]), and tear lipid layer interference (graded 1–5, as assessed via a DR-1 tear interference camera [Kowa Co., Nagoya, Japan] according to the grading proposed by Yokoi et al. [8]), were evaluated.

### Evaluation of efficacy

**Fig. 2:**
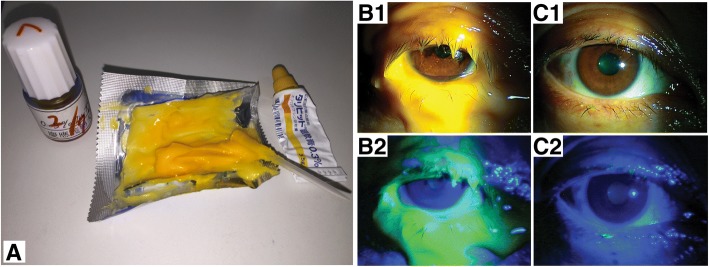
Efficacy evaluation based on the washing rate of fluorescein-stained 0.3% Tarivid ointment from lids. Efficacy is determined according to the extent of the staining level of both the upper and lower lids, including the lid margins after lid hygiene. Preparation of the fluorescein-stained 0.3% Tarivid ointment is shown (a). Lid staining before/after washout under normal light (b1/c1) and blue light using a cobalt blue filter (b2/c2)

The efficacy of each method was determined based on the washout rate of fluorescein-stained 0.3% Tarivid ophthalmic ointment (Ofloxacin ointment), which was fully applied to the eyelids, including the lid margins (Fig. 2a, b1 and b2). Post-wiping scores were determined for the ointment remaining on the eyelids under microscopic view illuminated by blue light (graded 0–6 based on Table 2, Fig. 2c1 and c2) just after performing each of the four lid hygiene methods described above.

**Table 2:** Classification of fluorescein-stained 0.3% Tarivid ointment staining score of the eyelid. Aggregate scores (0–6) of each of the upper (0–3) and lower lids (0–3), including the lid margins, are used in this study

0	1	2	3
No staining	Partial staining (low)	Partial staining (high)	Full staining

### Statistical analysis

We used Wilcoxon signed-rank tests to analyze all items evaluated in this study. When the efficacies of the 4 lid hygiene methods (water alone, Eye Shampoo, Eye Brush, and both Eye Shampoo and the Eye Brush) were compared, the results were interpreted by taking the multiple tests for significance into account since there were 6 comparison pairs. The Bonferroni method [9, 10] was used to correct for multiple comparisons, and a difference was considered significant when the *P*-value was less than 0.05/6 = 0.0083. When the efficacy of 3 lid hygiene methods, i.e., water alone, Eye Shampoo, and Eye Shampoo with the Eye Brush, was compared in one-tailed hypothesis (water < Eye Shampoo < Eye Shampoo and Eye Brush) tests, there were 2 comparison pairs, and according to the Bonferroni correction, a difference was considered significant when the *P*-value was less than 0.05/2 = 0.025. Similarly, when comparing the efficacy of water alone, the Eye Brush, and Eye Shampoo with the Eye Brush in one-tailed hypothesis (water < Eye Brush < Eye Shampoo and Eye Brush) tests, there were 2 comparison pairs, and according to the Bonferroni correction, a difference was considered significant when the P-value was less than 0.05/2 = 0.025. We did not combine the scores from all tests to arrive at one composite score and calculate significant differences. Scores from different investigative modalities were not combined in this study.

This prospective clinical pilot comparative study was registered with the University Hospital Medical Information Network Clinical Trial Registry in Japan (UMIN000016905). We adhered to the tenets of the Declaration of Helsinki, and the ethics committee of the Keio University School of Medicine approved the protocol.

## Results

### Evaluation of safety

**Fig. 3:**
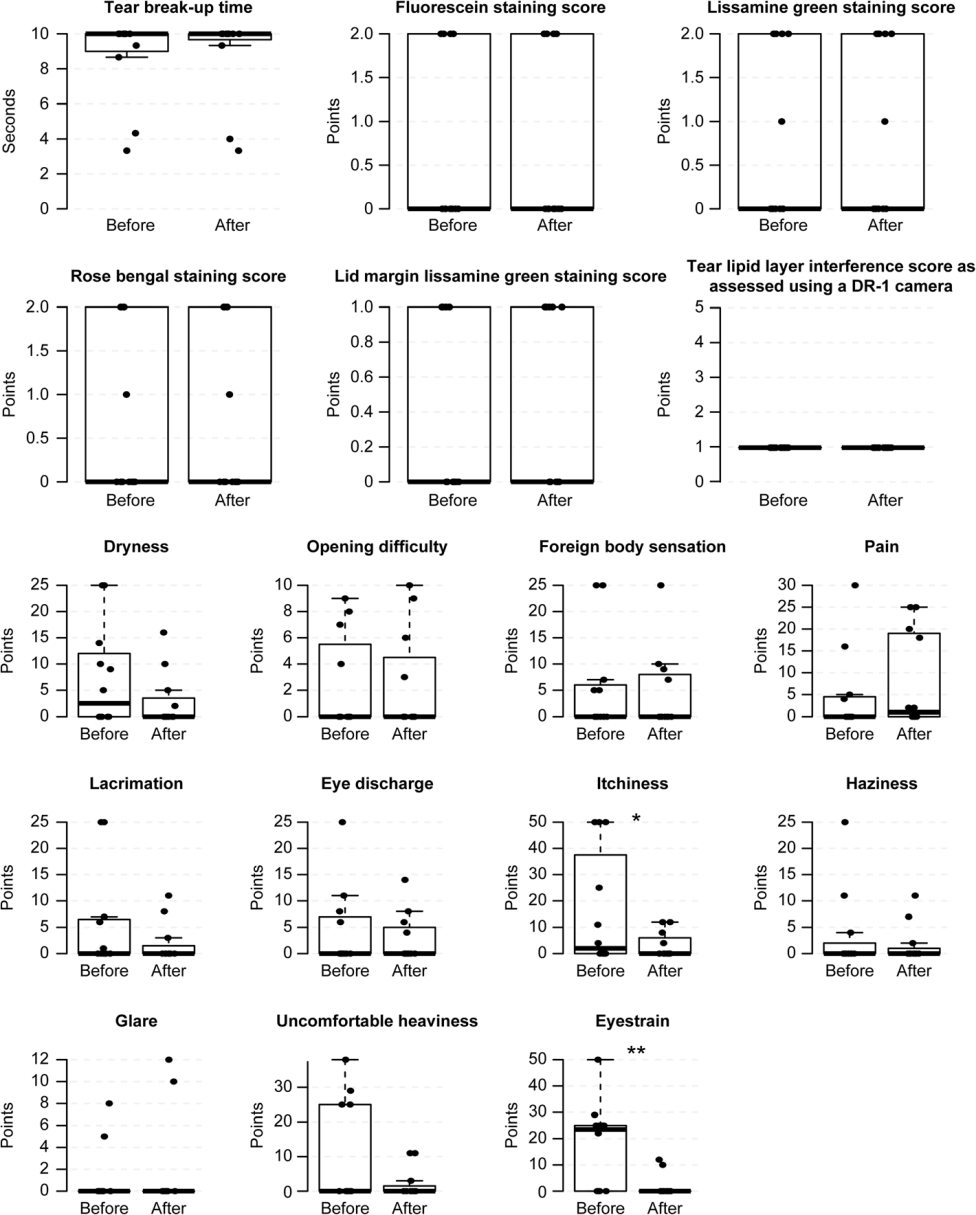
Results before/after wiping the lid margins using tap water in subjects with normal meibomian glands. In the box-and-whisker plots, the bottom of the box indicates the first quartile, and the top of the box indicates the third quartile. The band inside the box represents the median. To highlight suspected outliers, the upper whisker is set as the maximum or the third quartile+ 1.5 × IQR. The lower whisker indicates the minimum or the first quartile-1.5 × IQR. The bee swarm plot is a one-dimensional scatter plot with non-overlapping points. Note that because the placement of the dots in figures is randomly determined in the bee swarm plot, the dots are sometimes merged. ** Significant improvement; *P* < 0.05 and * Noted difference; *P* < 0.1 are demonstrated

**Fig. 4:**
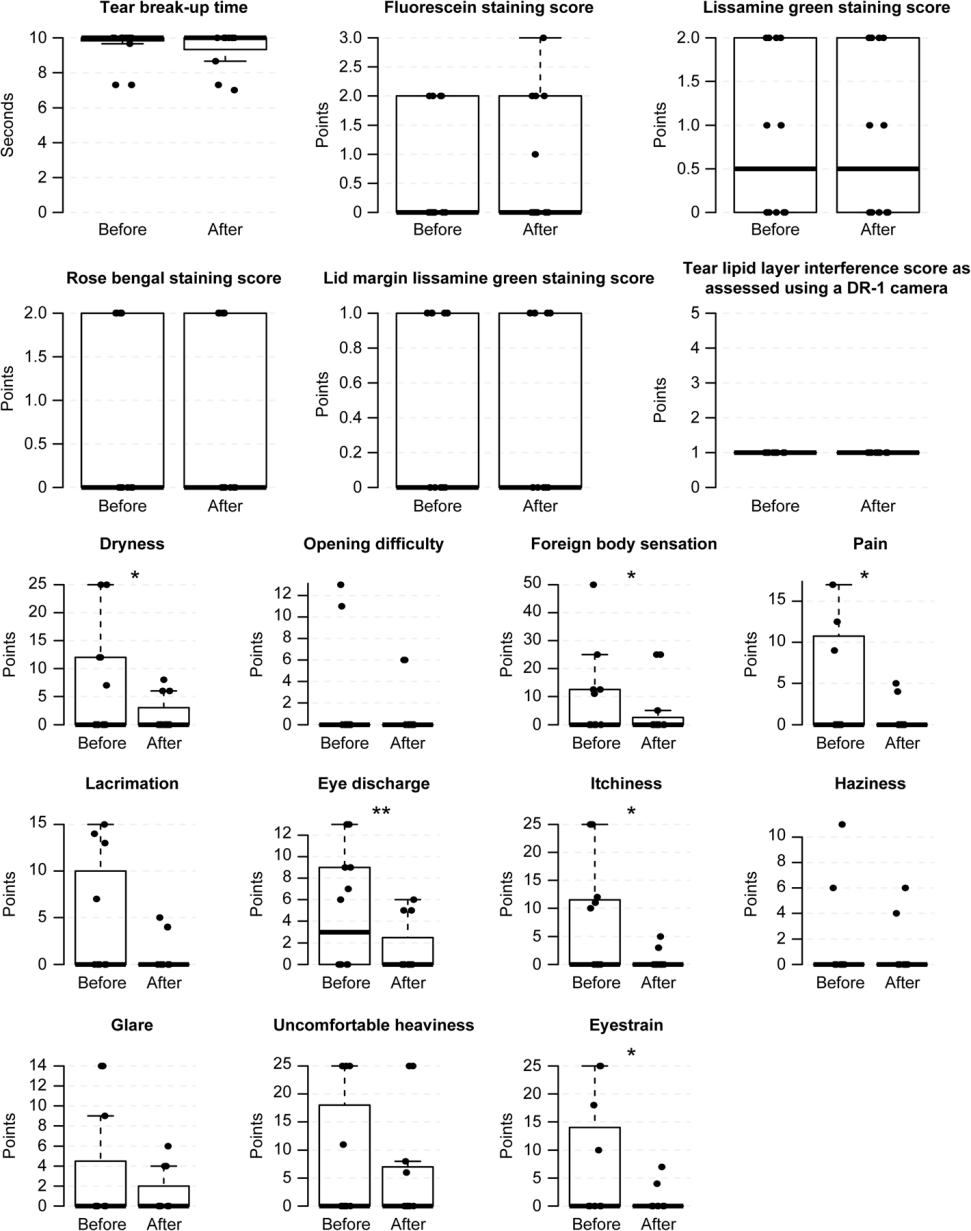
Results before/after wiping the lid margins using Eye Shampoo in subjects with normal meibomian glands. In the box-and-whisker plots, the bottom of the box indicates the first quartile, and the top of the box indicates the third quartile. The band inside the box represents the median. To highlight suspected outliers, the upper whisker is set as the maximum or the third quartile+ 1.5 × IQR. The lower whisker indicates the minimum or the first quartile-1.5 × IQR. The bee swarm plot is a one-dimensional scatter plot with non-overlapping points. Note that because the placement of the dots in figures is randomly determined in the bee swarm plot, the dots are sometimes merged. ** Significant improvement; *P* < 0.05 and * Noted difference; *P* < 0.1 are demonstrated

**Fig. 5:**
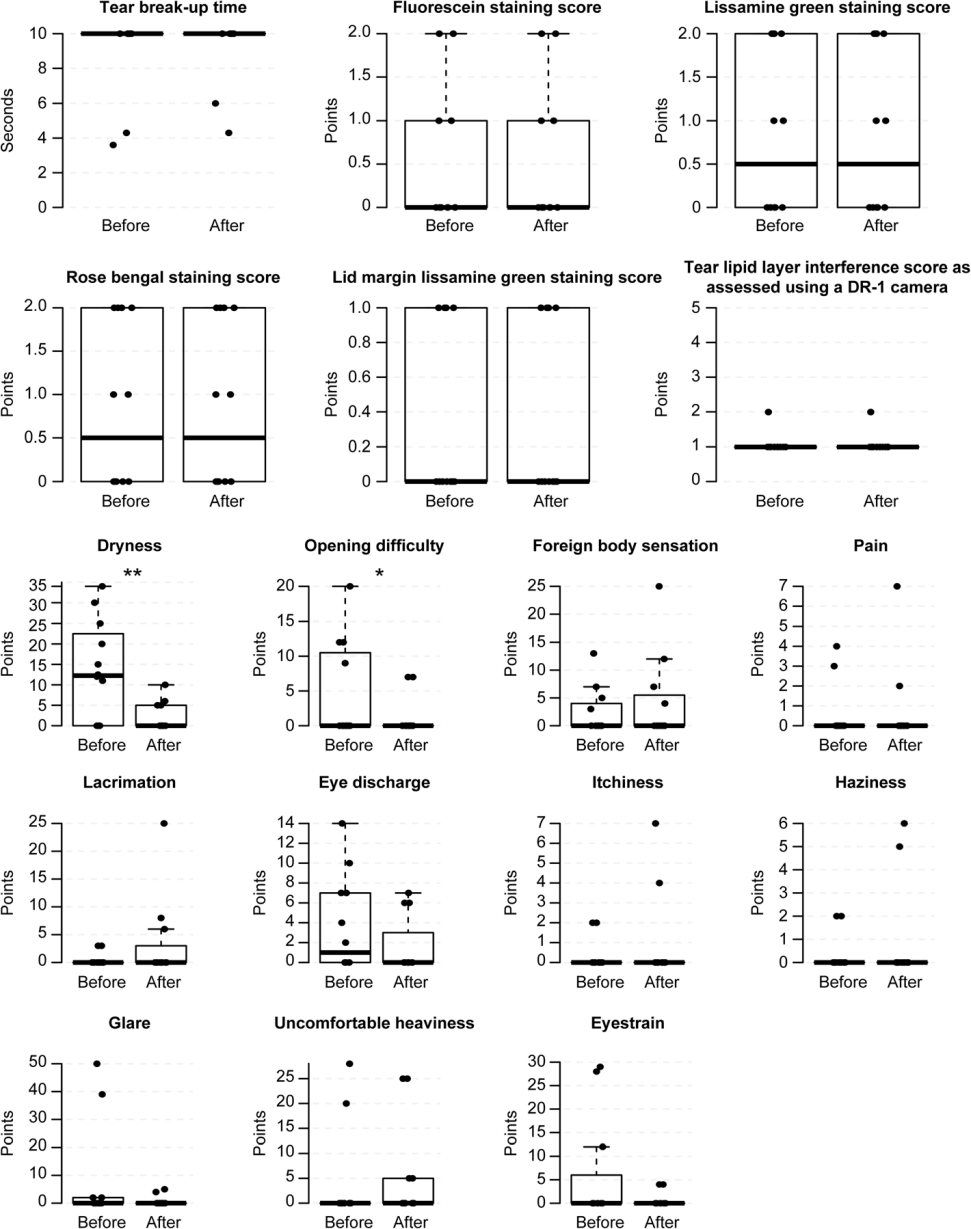
Results before/after wiping the lid margins using Eye Brush in subjects with normal meibomian glands. In the box-and-whisker plots, the bottom of the box indicates the first quartile, and the top of the box indicates the third quartile. The band inside the box represents the median. To highlight suspected outliers, the upper whisker is set as the maximum or the third quartile+ 1.5 × IQR. The lower whisker indicates the minimum or the first quartile-1.5 × IQR. The bee swarm plot is a one-dimensional scatter plot with non-overlapping points. Note that because the placement of the dots in figures is randomly determined in the bee swarm plot, the dots are sometimes merged. ** Significant improvement; *P* < 0.05 and * Noted difference; *P* < 0.1 are demonstrated

**Fig. 6:**
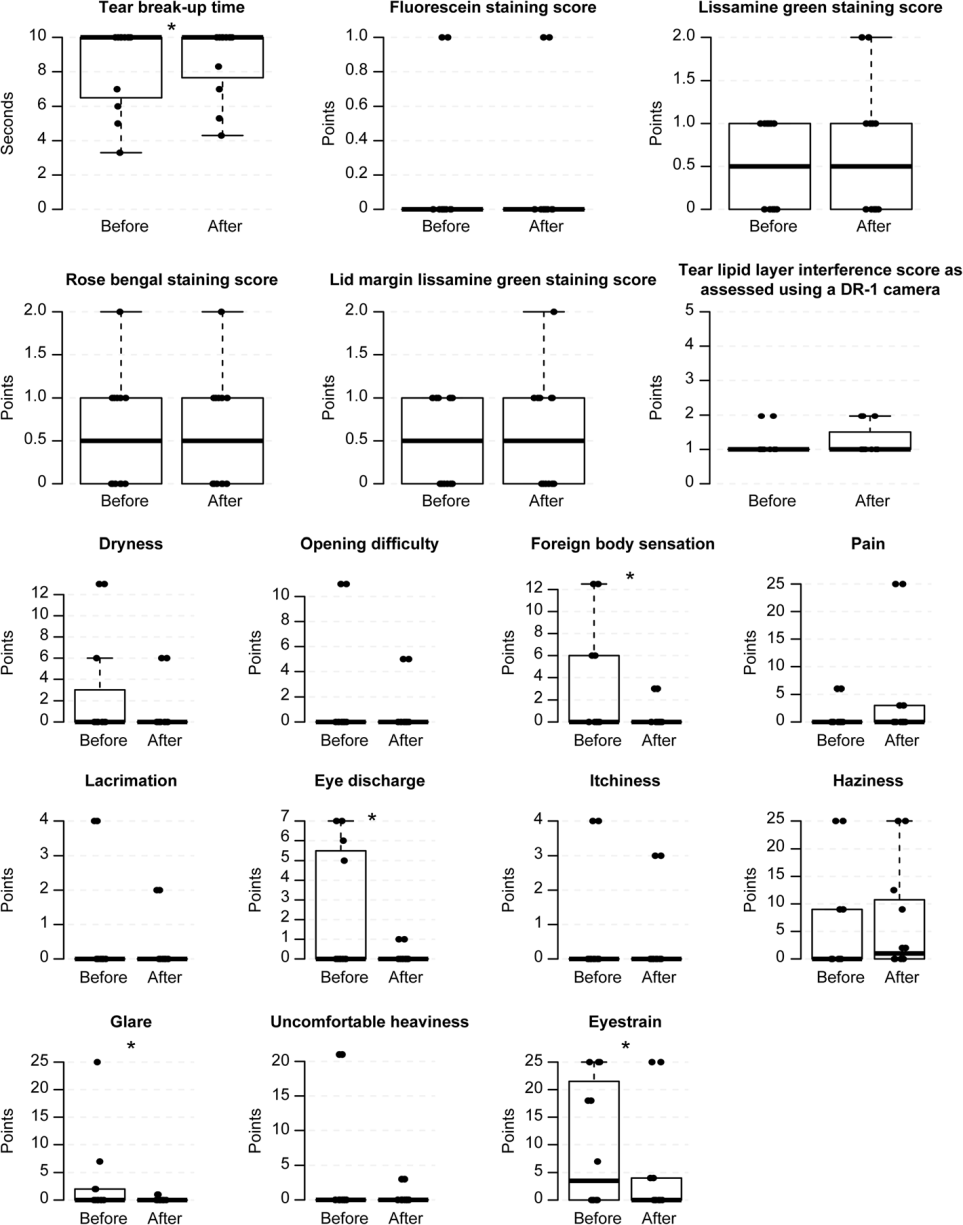
Results before/after wiping the lid margins using both Eye Shampoo and the Eye Brush. In the box-and-whisker plots, the bottom of the box indicates the first quartile, and the top of the box indicates the third quartile. The band inside the box represents the median. To highlight suspected outliers, the upper whisker is set as the maximum or the third quartile+ 1.5 × IQR. The lower whisker indicates the minimum or the first quartile-1.5 × IQR. The bee swarm plot is a one-dimensional scatter plot with non-overlapping points. Note that because the placement of the dots in figures is randomly determined in the bee swarm plot, the dots are sometimes merged. * Noted difference; *P* < 0.1 is demonstrated

**Fig. 7:**
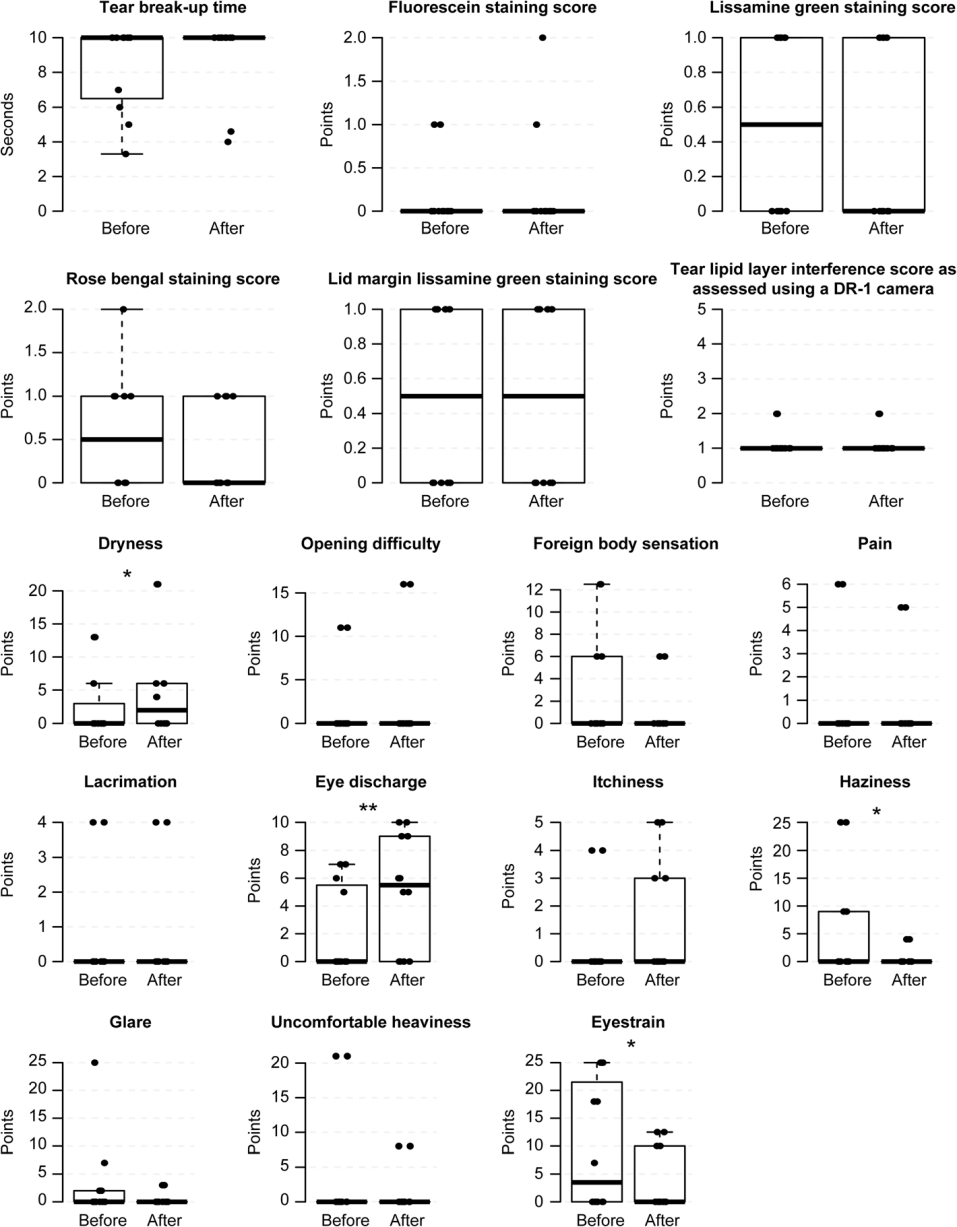
Results after using both Eye Shampoo and the Eye Brush twice daily for 1 month. In the box-and-whisker plots, the bottom of the box indicates the first quartile, and the top of the box indicates the third quartile. The band inside the box represents the median. To highlight suspected outliers, the upper whisker is set as the maximum or the third quartile+ 1.5 × IQR. The lower whisker indicates the minimum or the first quartile-1.5 × IQR. The bee swarm plot is a one-dimensional scatter plot with non-overlapping points. Note that because the placement of the dots in figures is randomly determined in the bee swarm plot, the dots are sometimes merged. ** Significant improvement; *P* < 0.05 and * Noted difference; *P* < 0.1 are demonstrated

There was no significant deterioration in TBUT, corneal and conjunctival staining scores, lid-margin staining scores or DR-1 scores after any method (Figs. 3, 4, 5, 6 and 7, Additional file 1, 2, 3 and 4). Eyestrain VAS scores significantly decreased with tap water (23.5 (0–25) to 0 (0–0), *P* = 0.020) (Fig. 3, Additional file 1), eye discharge significantly decreased with Eye Shampoo use (3 (0–9) to 0 (0–1.25), *P* = 0.036) (Fig. 4, Additional file 2), and dryness decreased with Eye Brush use (12.25 (0–21.25) to 0 (0–5), *P* = 0.014) (Fig. 5, Additional file 3). Sensations of eye discharge significantly increased after 1 month of using both the Eye Brush and Eye Shampoo (0 (0–5.25) to 5.5 (0–9), *P* = 0.042) (Fig. 6, Additional file 4).

### Evaluation of efficacy

**Fig. 8:**
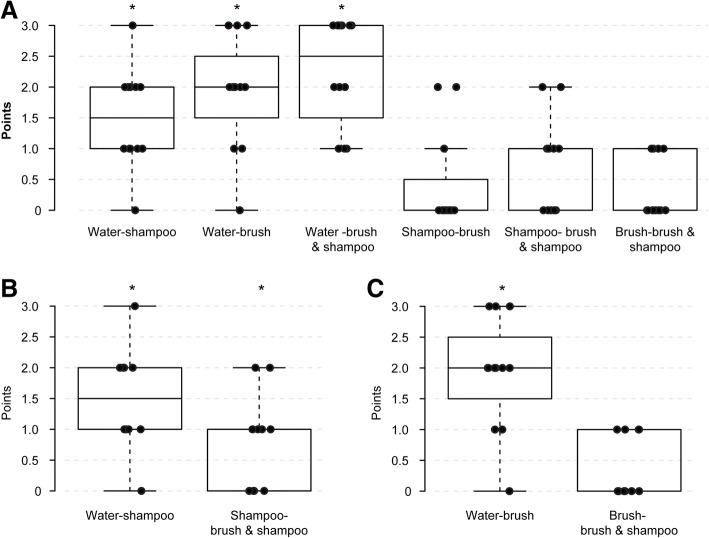
**a-c** Efficacy study results based on the lid staining scores. Fluorescein-stained 0.3% Tarivid ointment was used for grading (Table 2) after wiping the lid margins using tap water alone, Eye Shampoo, the Eye Brush, or both Eye Shampoo and the Eye Brush in subjects with normal meibomian glands. The wiping efficacies of each of the four lid hygiene methods were compared (**a**). The efficacy using both Eye Shampoo and the Eye Brush was compared with that using Eye Shampoo only (**b**) or the Eye Brush only (**c**) in one-tailed hypothesis (water < Eye Shampoo < Eye Shampoo and Eye Brush, water < Eye Brush < Eye Shampoo and Eye Brush) tests. In the box-and-whisker plots, the bottom of the box indicates the first quartile, and the top of the box indicates the third quartile. The band inside the box represents the median. To highlight suspected outliers, the upper whisker is set as the maximum or the third quartile+ 1.5 × IQR. The lower whisker indicates the minimum or the first quartile-1.5 × IQR. The bee swarm plot is a one-dimensional scatter plot with non-overlapping points. Note that because the placement of the dots in figures is randomly determined in the bee swarm plot, the dots are sometimes merged. * Significant improvement; *P* < 0.0083 [0.05/6 = 0.0083, Bonferroni correction] (**a**) and * Significant improvement; *P* < 0.025 [0.05/2 = 0.025, Bonferroni correction] (**b**, **c**) are demonstrated

Wiping efficacy when using Eye Shampoo, the Eye Brush, or both Eye Shampoo and the Eye Brush was significantly greater than that when using tap water alone (two-tailed hypothesis tests and no-correction, *P* = 0.00319, 0.00319, 0.00213, respectively) (Fig. 8a, Additional file 5). Using both products showed a significantly higher efficacy than using Eye Shampoo alone (one-tailed hypothesis tests and no-correction, *P* = 0.00883) (Fig. 8b, Additional file 6), but efficacy was not significantly greater than that when using the Eye Brush alone (one-tailed hypothesis tests and no-correction, *P* = 0.03593) (Fig. 8c, Additional file 7).

## Discussion

In this comparative clinical pilot study, we evaluated the safety and efficacy of wiping the lid margins with lid hygiene shampoo using the “Eye Brush,” a lid hygiene brush prototype, in subjects with normal meibomian glands. Based on our results, wiping lid margins using the Eye Brush was safe and enhanced the cleansing power of Eye Shampoo. Eyestrain VAS scores significantly decreased with tap water, eye discharge significantly decreased with Eye Shampoo use, and dryness decreased with use of the Eye Brush. However, the sensation of eye discharge significantly increased after 1 month of using both the Eye Brush and Eye Shampoo. Considering that no deterioration other than the sensation of eye discharge was observed, this increase could be the result of positive metabolic activation and/or an improved awareness of the importance of lid hygiene, but the reason for this observation must be investigated in a future study involving more subjects.

According to a previous study, washing eyes with tap water alone could cause deterioration of the ocular surface [11]. This concern might reduce the frequency of lid hygiene routines using tap water. Therefore, we performed this safety and efficacy study to evaluate the effects of wiping lid margins with tap water alone or in combination with lid hygiene shampoo in subjects with normal meibomian glands. We asked the subjects to keep their eyes lightly closed while washing their eyes for 30 s. This protocol was demonstrated to be both safe and efficacious, i.e., significant subjective improvements were observed.

In this study, we used lid hygiene shampoo (Eye Shampoo), which was created under the assumption that a mixture of the shampoo and tap water could accidentally get into the eyes. This shampoo is adjusted to the pH and osmolarity of normal tears and includes certain components that are beneficial to the ocular surface, such as anti-inflammatory and moisturizing substances (see Table 1 for information on Eye Shampoo).

As we hypothesized before the study regarding the potential effect of the Eye Brush on lid hygiene, the results show that it was effective not only when used alone but also in combination with Eye Shampoo, significantly enhancing the cleansing power of Eye Shampoo.

It is desirable that eye brushes are gentle on the eyes and have high cleaning efficacy. Eye brushes should be minimally stimulating and not cause vibration-induced retinal and posterior vitreous detachment or wrinkle formation on the skin. First, we prepared a brush with certain safety and cleaning efficacy properties as a prototype (patent pending, refer to the application) as shown in this study. Currently, we are engaged in joint development with Japanese Bio Mechanics with the aim of developing a brush that employs ultrasound, which is more effective and gentle for the eyes, while appropriately everting the eyelid margin and retaining a massaging effect on the meibomian glands. We are planning to perform a pilot study with this new brush in the future to provide a brush with increased safety and cleaning efficacy that minimizes vibration (amplitude x vibration frequency) of the eyes.

There are limitations in this study. First, although we performed a controlled study on subjects with normal meibomian glands comparing the result of different types of lid hygiene, both eyes of each subject should not have been included as targets of evaluation since the eyes of the same subjects could be similar. However, comparing the characteristic of lid hygiene with an eye brush, both eyelids are definitely cleansed in a different way, i.e., some subjects use their brush with right hands, others their left and some both. Thus, including both eyes in the evaluation could be reasonable. Second, in our studies, no subject or investigator masking was employed. This lack of masking might cause placebo effects on the results. Furthermore, avoiding subjective scoring without individual interpretation requires more than one investigator to see the eye and grade the various tests. Third, we diagnosed meibomian glands as normal using the criteria suggested by a previous report or the International Workshop on Meibomian Gland Dysfunction (Additional file 8 for information on the diagnosis of MGD) [12, 13]. However, there are no globally accepted absolute diagnostic criteria, and we made diagnoses according to our own standards. This issue could result in differences in the enrollment of subjects with normal meibomian glands among different research groups. Finally, due to the limited number of patients, the conclusion is also limited. However, using the same eyes for all interventions in this study rather than recruiting more subjects and using one procedure for one set of subjects could be reasonable in that the different eyes could respond to the same procedure in a different way due to their own innate characteristics. In any case, a larger study is necessary to support the results.

## Conclusions

Wiping lid margins using Eye Brush was safe and enhanced the cleansing power of Eye Shampoo. In conclusion, a lid-margin cleansing routine with the Eye Brush and using lid hygiene shampoo, although still requiring future development, could become an ideal healthcare solution for daily lid hygiene.

## Additional files

Additional file 1: Supplementary Table for Fig. 3. (PDF 54 kb)

Additional file 2: Supplementary Table for Fig. 4. (PDF 54 kb)

Additional file 3: Supplementary Table for Fig. 5. (PDF 54 kb)

Additional file 4: Supplementary Table for Fig. 6, 7. (PDF 55 kb)

Additional file 5: Supplementary Table for Fig. 8a. (PDF 50 kb)

Additional file 6: Supplementary Table for Fig. 8b (PDF 50 kb)

Additional file 7: Supplementary Table for Fig. 8c (PDF 49 kb)

Additional file 8: The criteria for the diagnosis of MGD suggested by a previous report and the International Workshop on Meibomian Gland Dysfunction. (PNG 699 kb)

## Abbreviations

MGD: Meibomian gland dysfunction
TBUT: Tear break-up time
VAS: Visual analog scale
