# Supplementary material for: Safety and efficacy of wiping lid margins with lid hygiene shampoo using the “eye brush”, a novel lid hygiene item, in healthy subjects: a pilot study
Source: BMC Ophthalmol. 2019 Feb 4;19:41. doi: 10.1186/s12886-019-1052-y (PMC6360667; doi:10.1186/s12886-019-1052-y)
Supplement: Supplementary file 1 — Supplementary Table for Fig. 3. (PDF 54 kb) [file 12886_2019_1052_MOESM1_ESM.pdf]

Additional file 1 for Supplementary Table for Figure 3

Results before/after wiping the lid margins using tap water alone in healthy subjects.

Parameter	Before		After		P-value
	Median	1st Q, 3rd Q	Median	1st Q, 3rd Q	
BUT	10	9.1625,10	10	9.8325,10	0.850
Fluorescein	0	0,2	0	0,2	NA
Lissamine green	0	0,2	0	0,2	NA
Rose bengal	0	0,2	0	0,2	NA
Lid	0	0,1	0	0,1	1.000
DR-1	1	1,1	1	1,1	NA
Dryness	2.5	0,11	0	0,2.75	0.197
Opening difficulty	0	0,4.75	0	0,3.75	0.854
Foreign body sensation	0	0,5.5	0	0,7.5	1.000
Pain	0	0,4.25	1	0,18.5	0.528
Lacrimation	0	0,6.25	0	0,0.75	0.785
Eye discharge	0	0,6.5	0	0,4.5	0.584
Itchiness	2	0,31.25	0	0,5	0.093*
Haziness	0	0,1	0	0,0.5	0.855
Glare	0	0,0	0	0,0	0.371
Uncomfortable heaviness	0	0,25	0	0,0.75	0.104
Eyestrain	23.5	0,25	0	0,0	0.020**

** Significant improvement; $P < 0.05$

* Noted difference; $P < 0.1$
