# Supplementary material for: Safety and efficacy of wiping lid margins with lid hygiene shampoo using the “eye brush”, a novel lid hygiene item, in healthy subjects: a pilot study
Source: BMC Ophthalmol. 2019 Feb 4;19:41. doi: 10.1186/s12886-019-1052-y (PMC6360667; doi:10.1186/s12886-019-1052-y)
Supplement: Supplementary file 2 — Supplementary Table for Fig. 4. (PDF 54 kb) [file 12886_2019_1052_MOESM2_ESM.pdf]

Additional file 2 for Supplementary Table for Figure 4

Results before/after wiping the lid margins using Eye Shampoo alone in healthy subjects.

Parameter					
Parameter<br>BUT	Before		After		P-value
	Median	1st Q, 3rd Q	Median	1st Q, 3rd Q	
Fluorescein	10	9.915,10	10	9.665,10	0.789
Lissamine green	0	0,2	0	0,2	0.346
Rose bengal	0.5	0,2	0.5	0,2	NA
Lid	0	0,2	0	0,2	NA
DR-1	0	0,1	0	0,1	NA
Dryness	1	1,1	1	1,1	NA
Opening difficulty	0	0,12	0	0,1.5	0.058*
Foreign body sensation	0	0,0	0	0,0	0.371
Pain	0	0,12.5	0	0,1.25	0.098*
Lacrimation	0	0,9.875	0	0,0	0.098*
Eye discharge	0	0,8.5	0	0,0	0.100
Itchiness	3	0,9	0	0,1.25	0.036**
Haziness	0	0,11.25	0	0,0	0.058*
Glare	0	0,0	0	0,0	0.371
Uncomfortable heaviness	0	0,2.25	0	0,1	0.174
Eyestrain	0	0,14.5	0	0,6.5	0.371
	0	0,12	0	0,0	0.098*

** Significant improvement; P < 0.05

* Noted difference; P < 0.1
