# Supplementary material for: Safety and efficacy of wiping lid margins with lid hygiene shampoo using the “eye brush”, a novel lid hygiene item, in healthy subjects: a pilot study
Source: BMC Ophthalmol. 2019 Feb 4;19:41. doi: 10.1186/s12886-019-1052-y (PMC6360667; doi:10.1186/s12886-019-1052-y)
Supplement: Supplementary file 3 — Supplementary Table for Fig. 5. (PDF 54 kb) [file 12886_2019_1052_MOESM3_ESM.pdf]

Additional file 3 for Supplementary Table for Figure 5

Results before/after wiping the lid margins using the Eye Brush alone in healthy subjects.

Parameter	Before		After		P-value
	Median	1st Q, 3rd Q	Median	1st Q, 3rd Q	
BUT	10	10,10	10	10,10	1.000
Fluorescein	0	0,1	0	0,1	NA
Lissamine green	0.5	0,2	0.5	0,2	NA
Rose bengal	0.5	0,2	0.5	0,2	NA
Lid	0	0,1	0	0,1	NA
DR-1	1	1,1	1	1,1	NA
Dryness	12.25	0,21.25	0	0,5	0.014**
Opening difficulty	0	0,9.75	0	0,0	0.098*
Foreign body sensation	0	0,3.5	0	0,4.75	0.590
Pain	0	0,0	0	0,0	1.000
Lacrimation	0	0,0	0	0,1.5	0.181
Eye discharge	1	0,7	0	0,1.5	0.295
Itchiness	0	0,0	0	0,0	0.371
Haziness	0	0,0	0	0,0	0.371
Glare	0	0,2	0	0,0	0.584
Uncomfortable heaviness	0	0,0	0	0,5	0.581
Eyestrain	0	0,3	0	0,0	0.181

** Significant improvement; $P < 0.05$

* Noted difference; $P < 0.1$
