# Supplementary material for: Safety and efficacy of wiping lid margins with lid hygiene shampoo using the “eye brush”, a novel lid hygiene item, in healthy subjects: a pilot study
Source: BMC Ophthalmol. 2019 Feb 4;19:41. doi: 10.1186/s12886-019-1052-y (PMC6360667; doi:10.1186/s12886-019-1052-y)
Supplement: Supplementary file 4 — Supplementary Table for Fig. 6, 7. (PDF 55 kb) [file 12886_2019_1052_MOESM4_ESM.pdf]

Additional file 4 for Supplementary Table for Figure 6, 7

Results before/after wiping the lid margins using both Eye Shampoo and the Eye Brush. Results after using both twice daily for 1 month are also demonstrated.

Parameter	Before		After		1 M		P-value	
	Median	1st Q, 3rd Q	Median	1st Q, 3rd Q	Median	1st Q, 3rd Q	Before-After	Before-1 M
BUT	10	6.75,10	10	7.975,10	10	10,10	0.098*	0.201
Fluorescein	0	0,0	0	0,0	0	0,0	NA	1.000
Lissamine green	0.5	0,1	0.5	0,1	0	0,1	0.346	0.346
Rose bengal	0.5	0,1	0.5	0,1	0	0,1	NA	0.149
Lid	0.5	0,1	0.5	0,1	0.5	0,1	1.000	NA
DR-1	1	1,1	1	1,1.25	1	1,1	1.000	NA
Dryness	0	0,1.5	0	0,0	2	0,6	0.174	0.058*
Opening difficulty	0	0,0	0	0,0	0	0,0	0.346	0.346
Foreign body sensation	0	0,6	0	0,0	0	0,0	0.095*	0.346
Pain	0	0,0	0	0,3	0	0,0	0.577	0.346
Lacrimation	0	0,0	0	0,0	0	0,0	0.346	NA
Eye discharge	0	0,5.25	0	0,0	5.5	0,9	0.089*	0.042**
Itchiness	0	0,0	0	0,0	0	0,3	0.346	0.577
Haziness	0	0,9	1	0,9.875	0	0,0	0.581	0.095*
Glare	0	0,2	0	0,0	0	0,0	0.098*	0.581
Uncomfortable heaviness	0	0,0	0	0,0	0	0,0	0.346	0.346
Eyestrain	3.5	0,19.75	0	0,4	0	0,10	0.098*	0.058*

** Significant improvement; $P < 0.05$

* Noted difference; $P < 0.1$
