# Supplementary material for: Safety and efficacy of wiping lid margins with lid hygiene shampoo using the “eye brush”, a novel lid hygiene item, in healthy subjects: a pilot study
Source: BMC Ophthalmol. 2019 Feb 4;19:41. doi: 10.1186/s12886-019-1052-y (PMC6360667; doi:10.1186/s12886-019-1052-y)
Supplement: Supplementary file 5 — Supplementary Table for Fig. 8a. (PDF 50 kb) [file 12886_2019_1052_MOESM5_ESM.pdf]

Additional file 5 for Supplementary Table for Figure 8A

Efficacy study results based on lid staining scores for fluorescein-stained 0.3% Tarivid ointment. The wiping efficacies of the four lid hygiene methods, i.e., wiping the lid margins using tap water alone, Eye Shampoo, the Eye Brush, or both Eye Shampoo and the Eye Brush, were compared.

Method 1	Method 2	Wilcoxon signed-rank test
water	eye shampoo	0.00319*
water	lid margin brush	0.00319*
water	lid margin brush & eye shampoo	0.00213*
eye shampoo	lid margin brush	0.17357
eye shampoo	lid margin brush & eye shampoo	0.01766
lid margin brush	lid margin brush & eye shampoo	0.07186

Two-tailed hypothesis tests and no-correction

* Significant improvement; $P < 0.0083$ [0.05/6=0.0083, Bonferroni correction]
