# Supplementary material for: Safety and efficacy of wiping lid margins with lid hygiene shampoo using the “eye brush”, a novel lid hygiene item, in healthy subjects: a pilot study
Source: BMC Ophthalmol. 2019 Feb 4;19:41. doi: 10.1186/s12886-019-1052-y (PMC6360667; doi:10.1186/s12886-019-1052-y)
Supplement: Supplementary file 7 — Supplementary Table for Fig. 8c (PDF 49 kb) [file 12886_2019_1052_MOESM7_ESM.pdf]

Additional file 7 for Supplementary Table for Figure 8C

Efficacy study results based on lid staining scores for fluorescein-stained 0.3% Tarivid ointment. Efficacy was compared in one-tailed hypothesis (1) water < Eye Shampoo < Eye Shampoo and Eye Brush, 2) water < Eye Brush < Eye Shampoo and Eye Brush) tests.

Method 1	Method 2	Wilcoxon signed-rank test
water	Eye Brush	0.00160*
Eye Brush	Eye Brush & Eye Shampoo	0.03593

One-tailed hypothesis tests and no-correction

* Significant improvement; P < 0.025 [0.05/2=0.025, Bonferroni correction]
