# Supplementary figures and images for: Safety and efficacy of wiping lid margins with lid hygiene shampoo using the “eye brush”, a novel lid hygiene item, in healthy subjects: a pilot study
Source: BMC Ophthalmol. 2019 Feb 4;19:41. doi: 10.1186/s12886-019-1052-y (PMC6360667; doi:10.1186/s12886-019-1052-y)

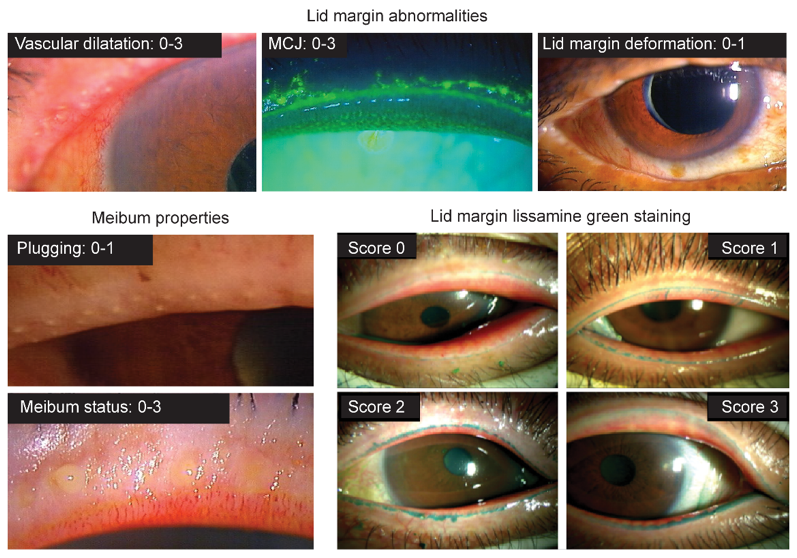

Supplement: Supplementary file 8 — The criteria for the diagnosis of MGD suggested by a previous report and the International Workshop on Meibomian Gland Dysfunction. (PNG 699 kb) [file 12886_2019_1052_MOESM8_ESM.png]
